# Synchrotron based phase contrast X-ray imaging combined with FTIR spectroscopy reveals structural and biomolecular differences in spikelets play a significant role in resistance to Fusarium in wheat

**DOI:** 10.1186/s12870-014-0357-5

**Published:** 2015-01-28

**Authors:** Rachid Lahlali, Chithra Karunakaran, Lipu Wang, Ian Willick, Marina Schmidt, Xia Liu, Ferenc Borondics, Lily Forseille, Pierre R Fobert, Karen Tanino, Gary Peng, Emil Hallin

**Affiliations:** Canadian Light Source Inc., 44 Innovation Boulevard, Saskatoon, SK S7N 2V3 Canada; National Research Council Canada, 110 Gymnasium Place, Saskatoon, SK S7N 0W9 Canada; University of Saskatchewan, 51 Campus Drive, Saskatoon, SK S7N 5A8 Canada; Saskatoon Research Centre, Agriculture and Agri-Food Canada, 107 Science Place, Saskatoon, SK S7N 0X2 Canada

## Abstract

**Background:**

Fusarium head blight (FHB), a scab principally caused by *Fusarium graminearum* Schw., is a serious disease of wheat. The purpose of this study is to evaluate the potential of combining synchrotron based phase contrast X-ray imaging (PCI) with Fourier Transform mid infrared (FTIR) spectroscopy to understand the mechanisms of resistance to FHB by resistant wheat cultivars. Our hypothesis is that structural and biochemical differences between resistant and susceptible cultivars play a significant role in developing resistance to FHB.

**Results:**

Synchrotron based PCI images and FTIR absorption spectra (4000–800 cm^−1^) of the floret and rachis from Fusarium-damaged and undamaged spikes of the resistant cultivar ‘Sumai3’, tolerant cultivar ‘FL62R1’, and susceptible cultivar ‘Muchmore’ were collected and analyzed. The PCI images show significant differences between infected and non-infected florets and rachises of different wheat cultivars. However, no pronounced difference between non-inoculated resistant and susceptible cultivar in terms of floret structures could be determined due to the complexity of the internal structures. The FTIR spectra showed significant variability between infected and non-infected floret and rachis of the wheat cultivars. The changes in absorption wavenumbers following pathogenic infection were mostly in the spectral range from 1800–800 cm^−1^. The Principal Component Analysis (PCA) was also used to determine the significant chemical changes inside floret and rachis when exposed to the FHB disease stress to understand the plant response mechanism. In the floret and rachis samples, PCA of FTIR spectra revealed differences in cell wall related polysaccharides. In the florets, absorption peaks for Amide I, cellulose, hemicellulose and pectin were affected by the pathogenic fungus. In the rachis of the wheat cultivars, PCA underlines significant changes in pectin, cellulose, and hemicellulose characteristic absorption spectra. Amide II and lignin absorption peaks, persistent in the rachis of Sumai3, together with increased peak shift at 1245 cm^−1^ after infection with FHB may be a marker for stress response in which the cell wall compounds related to pathways for lignification are increased.

**Conclusions:**

Synchrotron based PCI combined with FTIR spectroscopy show promising results related to FHB in wheat. The combined technique is a powerful new tool for internal visualisation and biomolecular monitoring before and during plant-microbe interactions to understand both the differences between cultivars and their different responses to disease stress.

**Electronic supplementary material:**

The online version of this article (doi:10.1186/s12870-014-0357-5) contains supplementary material, which is available to authorized users.

## Background

Fusarium head blight (FHB) caused by *Fusarium graminearum* is a serious fungal disease of wheat (*Triticum aestivum* L.), and barley in Canada and world-wide through which grain quality losses are induced by fungal trichotecene mycotoxins such as deoxynivalenol (DON) [1-4]. The People Republic of China, Canada, parts of southern Africa, Eastern Europe, South America, and the United States all have recorded FHB outbreaks and all countries continue to struggle with this destructive disease [5,6]. Bai and Shaner [7] reported that wheat scab can greatly reduce grain yield and quality. The infection starts during the crop flowering (anthesis) stage. The fungal spores germinate in the anthers, spread through the anthers into the florets, and into other florets through the nodes in the rachis. Symptoms of FHB in wheat include purple to black necrotic lesions, awn twisting and deformation, bleaching and tanning attributed to blight, and atrophy of the developing grain resulting in “tombstone” kernels [6,8]. Under prolonged warm and moist conditions, signs of the fungus can be seen as pink mycelilal masses on the surface of infected spikes [6]. Occasionally, rachis of a blighted head will be girdled to the loss of the entire spike. Symptoms of FHB on barley include isolated areas of tan to dark brown discoloration as well as evidence of water-soaking restricted to the initially infected inflorescence [6,9]. Other abiotic factors such as freezing damage (*Gaeumannomyces graminis* var. tritici) can mask classic FHB disease symptoms making disease evaluations difficult. Most often severity is recorded as the number of diseased florets over the total number of florets per spike. Counting the total number of florets on several individual spikes in replicated plots for many genotypes in more than one environment can be a daunting task even for a team of researchers. And finally, with intricate irrigation systems and the total number of person hours needed to score multiple genotypes, the cost of one FHB data point has been reported as six US dollars [10].

Fungicidal effect on FHB has been variable in different studies. Cultivar resistance, fungicide efficacy, timing, and pathogen aggressiveness are probably some of the reasons for the variable effect of fungicides on FHB [11,12]. Fungicide treatment and agricultural management practices only reduce the damage, but they cannot prevent yield and quality losses [13]. Effective chemical control of FHB has generally been inconsistent [14]. Lower levels of at most 70% effectiveness have been reported for fungicide control in field conditions for naturally infected wheat [15]. Glasshouse and field trials conducted to assess the efficacy of fungicides against FHB yielded conflicting results. A possible explanation for this finding is the complex interaction that may occur between fungicide, FHB, and others fungal colonizers in the plant [16]. In addition, the effectiveness of fungicides against FHB is influenced by complex interaction between rainfall, temperature, fungicide concentration, and the time of application [17]. The complex nature of wheat resistance to FHB makes it difficult to select for via conventional breeding [18]. Until now, no absolute FHB resistance encoded by single dominant resistance genes has been characterized in wheat. Consequently, it is difficult to implement Fusarium resistance into breeding programs. Two major types for resistance have been characterized. Type I resistance stops the pathogen at the level of penetration while type II resistance involves the inhibition of fungal spread within the infected node [19,20]. However, the implementation of quantitative trait loci associated with resistance into commercial wheat varieties is not very easy due to high costs. Unfortunately, most resistant germplasm is of exotic origin and possesses poor agronomic traits; inheritance of resistance is oligogenic to polygenic; and screening for resistance is environmentally biased, tedious, and expensive [21].

Understanding host-pathogen interactions is important for the rational development of disease resistant plant varieties. Previous studies have used electron, confocal, and light microscopy to determine structural differences between fungal resistant and susceptible cultivars of wheat and barley [1,22-24] . It has been concluded that structural and biochemical characteristics of rachis in resistant lines may play a key role in restricting the progression of FHB [23]. These laboratory based analytical methods are more destructive and chemically less sensitive than synchrotron based techniques. Therefore, we propose to combine the structural and spatially resolved compositional information between resistant and susceptible cultivars to develop a more complete understanding of fungal infection in crops.

The structural visualization is essential to fully understand the structure-function relationship of plants. The structural characteristics of plant parts have been studied using conventional and destructive microscopy techniques such as fluorescence and electron microscopy [23,25]. The results from these techniques are limited by sample preparation constraints and a large area (or number) of an intact plant part cannot be studied. The long term monitoring of a plant or plant part to understand the physiological changes and responses to biotic, abiotic, or nutritional stresses cannot be studied using the above mentioned destructive techniques.

The use of X-rays for agricultural applications started in the 1920s and 3D visualization of structures using X-rays was demonstrated in 1973 [26-28]. The X-rays from a synchrotron have unique properties such as high intensity and wavelength selectability compared to laboratory based X-ray machines. Therefore, synchrotron based X-ray imaging of plants are fast due to high intensity in a narrow bandwidth which reduces the radiation dose absorbed by the plants. The X-ray wavelengths can be easily tuned to image above soil plant parts or soil-root systems and it is possible to image low density materials like plants in great detail using Phase contrast X-ray imaging (PCI) technique [29,30].

Fourier transform mid infrared (FTIR) spectroscopy is a physico-chemical analytical technique that provides a snapshot of tissue metabolic composition at a specified period under diverse environments [31-33]. FTIR generates a spectrum by the vibrations of bonds within chemical functional groups that can be considered as a biochemical or metabolic “fingerprint” of the sample. By assessing the infrared absorption peak width, position, and intensity, the configuration of molecular functional assemblies in a sample can be evaluated [34]. In most cases, the structure of the plant biomass being already known, the absorption peaks of the molecular bonds can be found in the literature, and changes in some of these absorption peaks due to the presence of any plant stress can be easily detected [35]. Applying metabolomics techniques to plant pathology is a new approach, generally used as a complementary method to transcriptome and proteome analyses [33]. Recently, it was investigated in plant microbe interaction as powerful and rapid methods to elucidate structural and chemicals changes associated with fungal infection [33,36-39]. It has been previously demonstrated that the composition of plant cell walls varies significantly from one cell type to another, one species to another, and between accessions within species with 30% cellulose, 30% hemicellulose, 35% pectin and 1 to 5% structural proteins [40]. Apart from lignin and phenolic components which are known to play an important role in plant defense by forming a physical barrier or inducing the defense of the host [41], little is known about the degree to which the chemical composition of plant cell wall polysaccharides is a key factor in the outcome of the plant-pathogen interaction. Vorwerk et al. [42] reviewed all of important findings on the role of plant cell composition in disease resistance. In this context, the main objectives of this study were to determine: 1) the structural differences (in floret and rachis) between resistant and susceptible cultivars of wheat using PCI; and 2) the biochemical changes in floret and rachis of resistant and susceptible cultivars of wheat before and following fungal infection using FTIR spectroscopy. The use of PCI coupled with FTIR provides a novel approach to discover the resistance mechanisms of the host against FHB infection, traditionally analyzed by destructive microscopy.

## Results

### FHB disease severity of wheat cultivars

**Figure 1:**
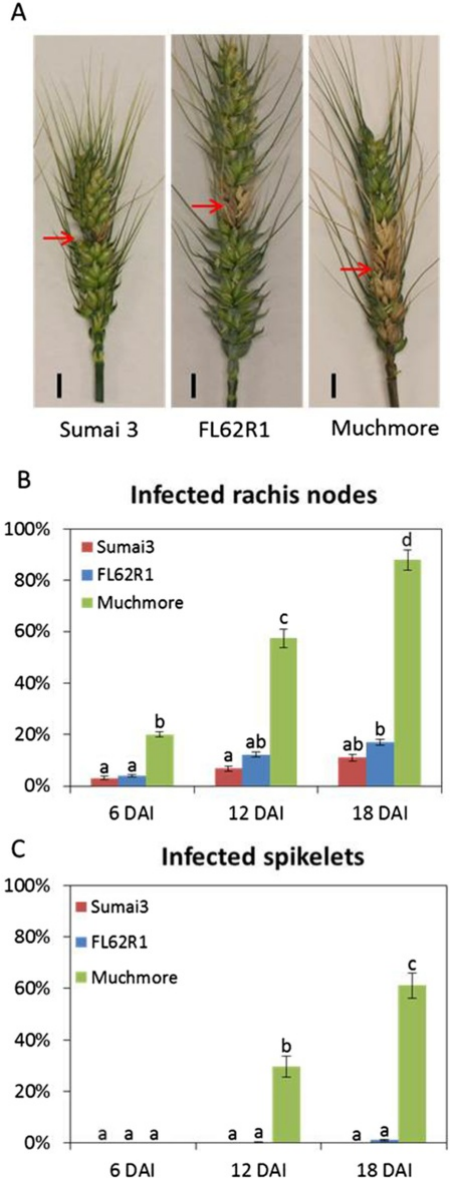
**Disease severity assessment after** ***Fg*** **-GFP** **point inoculation. (A)** *Fg*-GFP infected spikes at 18 DAI. Arrows indicate the site of point inoculation. **(B)** and **(C)** The percentage of infected rachis nodes and infected spikelet were scored at different time points. 40 heads and 20 plants per genotype were examined. A two-way ANOVA of data was performed at α = 0.01; treatments with common letters over the error bars are not significantly different from each other. Error bars represent standard error. This is one of three independent experiments with similar results.

FHB disease severity on three cultivars (Sumai3, FL62R1, and Muchmore) was assessed following point inoculation of a pair of middle spikelets with *Fusarium gramminearum* constitutively expressing GFP (Fg-GFP) (Figure 1A). Dark brown discoloration of inoculated spikelets was observed at 2 days after inoculation in all tested cultivars. Subsequently, the non-inoculated spikelets above and below the point of inoculation were bleached and started to dry up. Both dark brown and bleached spikelets were considered diseased. The number of infected rachis nodes and spikelets were scored at different time points. Among tested cultivars, Muchmore is most susceptible to Fg-GFP, which quickly colonized and started to spread in the rachis of this variety at an early time point (6 DAI) (Figure 1B). Eventually at 18 DAI, 90% rachis and 60% spikelets of Muchmore spike were diseased (Figure 1B and C). In contrast, Sumai3 and FL62R1 are more resistant to *Fg*-GFP compared to Muchmore. The spread of Fg-GFP was considerably reduced in the spikes of both cultivars (Figure 1B and C). The non-inoculated spikelets of Sumai3 and FL62R1 mostly remained green and healthy (Figure 1A). It is noteworthy that although Fg-GFP can spread to 1 or 2 rachis nodes in both cultivars (Figure 1B), it rarely spread into non-inoculated spikelets (Figure 1C), suggesting that rachilla, the tissue connecting between rachis and spikelet, may play important role to prevent Fg spreading into spikelet in the resistant cultivars. Infection progress of *Fg*-GFP was also observed under fluorescence microscope. Consistent with disease severity results in Figure 1, massive *Fg*-GFP were found and caused dark brown lesions in rachis and non-inoculated rachilla of susceptible cultivar, Muchmore (Additional file 1: Figure S1). However, few GFP signal and less disease necrotic lesions were observed in Sumai3 and FL62R1. Most tissue of resistant cultivars remained healthy and green, especially in rachilla.

### Phase contrast X-ray imaging of spikelets

**Figure 2:**
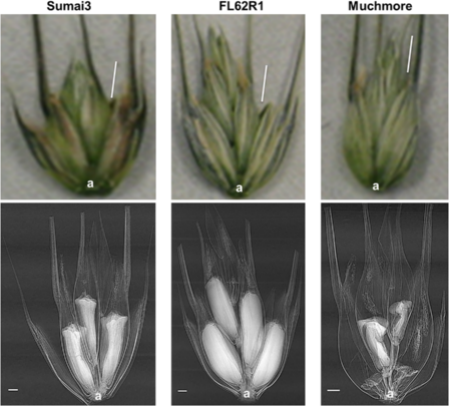
**Images of control and healthy florets in the spikelets of wheat cultivars using phase contrast X-ray imaging at 10 days after inoculation with FHB.** The florets were mounted on a kapton tape and X-ray images were recorded at 18 keV using a 8.75 μm resolution detector. Red inclined bar: the limit of the first fertile floret; **(a)**: the rachilla. Scale bars indicate 1 mm.

**Figure 3:**
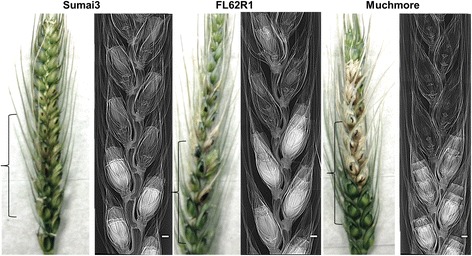
**Images of diseased and healthy florets in the spikelets of wheat cultivars using phase contrast X-ray imaging at 4 days after inoculation with FHB.** The spikelets were kept inside a 18 mm diameter falcon tube and X-ray images were recorded at 18 keV using a 8.75 μm resolution detector. Brackets indicate the imaged part of the wheat spike. Scale bars indicate 1 mm.

**Figure 4:**
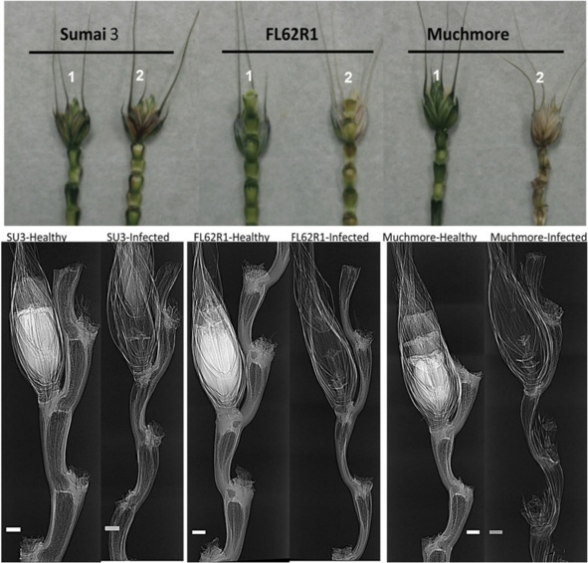
**Phase contrast X-ray images of healthy (1) and infected rachis (2) of wheat cultivars at 4 days after inoculation with FHB.** The spikelets were kept inside a 18 mm diameter falcon tube and X-ray images were recorded at 18 keV using a 8.75 μm resolution detector. Scale bars indicate 1 mm.

Synchrotron PCI has the potential to revolutionize the study of physiology and internal biomechanical structures in different plant samples. This non-destructive technique has the spatial and temporal resolution, penetrating power and sensitivity to soft tissue that is required to visualize the internal structure of living plants or animals on the scale from millimeters to microns [43]. In the current study, we have used this technique to compare the structural differences among resistant cultivar Sumai3, tolerant or Canadian resistant germplasm FL62R1, and moderately susceptible cultivar Muchmore. Scanned wheat heads of three cultivars that contain artificially infected and non-infected florets in the same spikelets with FHB at 4 DAI are shown in Figures 2 and 3. Differences in mass densities and phase contrast signals between healthy and infected spikelets were observed. Healthy florets appear in white colors filled with internal structures while infected ones are largely empty and transparent, perhaps due to loss of water and floret tissues. The high X-ray energy (18 keV) and the low resolution detector (8.75 μm) used here are not able to reveal any visible fungal structures such as mycelia of *Fusarium graminearum*. A total loss of cell viability in infected floret structures such as external and internal epidermis of glumes, and external and internal epidermis of anthers is revealed by the X-ray images (Figure 4). This phenomenon was more pronounced in Muchmore cultivar compared to the other two cultivars. The ovary in an infected Muchmore floret appears to be in a stressed state with the absence of anthers which may be destroyed by the fungus itself, indicating a loss of fertility in the infected spikelets of that cultivar.

To further elucidate resistance mechanisms and structural differences between three tested germplasms in response to FHB, florets were removed from the healthy and infected spikes of the wheat and rachis alone were imaged (Figure 4). In healthy rachis of different cultivars, phase contrast images show significant differences in the physical and internal structures of the rachises. The healthy rachis of Sumai3 is more transparent than that of FL62R1 and Muchmore near the floret base, indicating less internal structures and more cavitations in Sumai3. The internodes of the rachis joints in resistant cultivars are closed with a well-defined wall (visible bright line). Interestingly, form and thickness of edge of the rachis are different from cultivar to cultivar. In the presence of the fungus, the phase contrast X-ray images show that structures internal in rachis could be lost or altered. The cavitation (transparent area in phase contrast images and in which water movement occurs in the rachis) becomes thinner in infected rachises with FHB. These characteristic different structures in resistant cultivars compared to moderately resistant and susceptible ones may serve to limit the growth and spread of the fungal mycelium. The structural difference may also reduce the spread of fungal mycelial mass along with water flow within the rachis, which is considered to be one of the causes of spike blight symptoms.

### Mid infrared absorption spectroscopy

#### Biochemical changes in wheat floret

**Figure 5:**
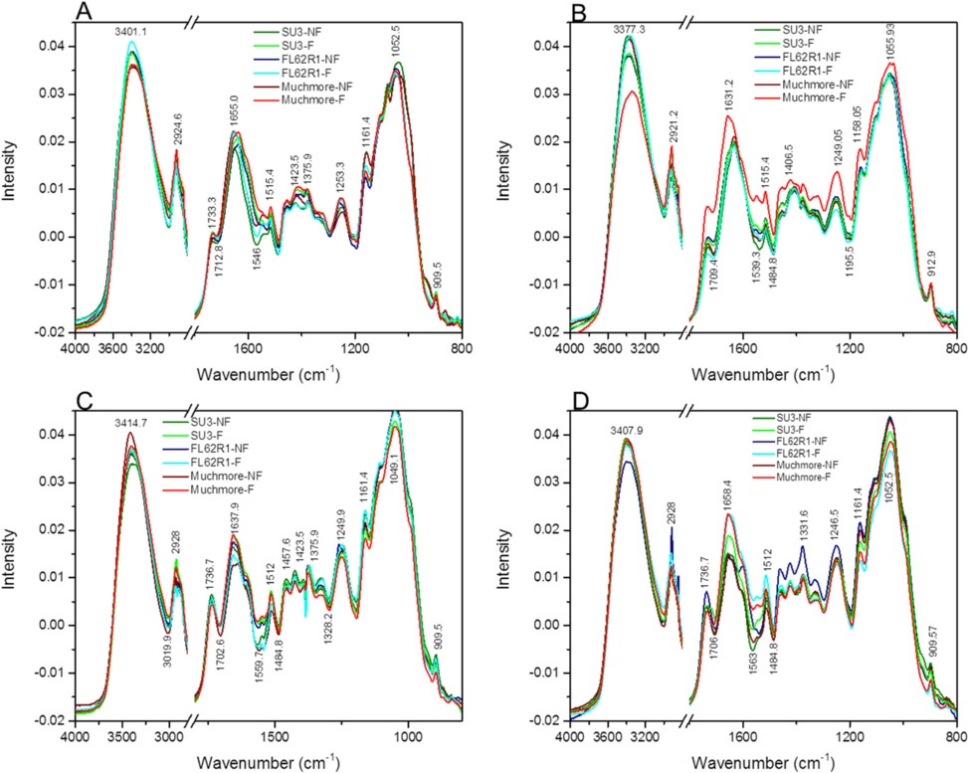
**Averaged triplicates of mid infrared absorbance spectra of wheat florets and rachis at 4 days after inoculation (A, C) and 10 days after inoculation (B, D) with FHB.** NF: non-infected, F: infected with Fusarium.

The mid infrared spectra of a chemical compound provide details of the fundamental vibrations of the groups of its component molecules. The IR spectrum of a biological sample is a weighted spectrum of individual chemical compounds present in that sample [44]. Obvious spectral differences in the mid infrared region (4000–800 cm^−1^) between healthy and diseased florets and rachis of wheat spikelets of different germplasms are shown in Figure 5. The summary of the characteristic peaks and their assignments in reference to previous findings are shown in the Additional file 2: Tables S1-S4 [33,37-39,45-47].

At 4 DAI, spectra of diseased and healthy floret of different wheat cultivars are shown in Figure 5A. Apart from the intense but unspecific stretching bands for OH groups (3394–3407 cm^−1^) and alkyl C-H groups (~2921 cm^−1^), the spectra showed a prominent peak with a maximum near 1049–1032 cm^−1^ attributable to C-O vibrations. For each cultivar, the groups of samples without inoculation (controls) were clearly differentiated from the samples inoculated with FHB. Fingerprint spectral regions showed intense peaks for carbonyl compounds C = O groups (1733 cm^−1^), C–H bending in alkyl groups (1420 cm^−1^), and presence (amide I at about 1655 cm^−1^) [48,49]. A well-defined pattern was obtained with characteristic peaks centered at about 1546–1566 and 1515 cm^−1^ for aromatic skeletal vibrations and additional peaks at 1420, 1375 and 1246 cm^−1^ that coincide with the different methoxyphenolic substitutions in the aromatic units of lignin [48,49]. In the resistant cultivar Sumai3, the relatively large shifts in characteristic peaks of amide III, cellulose, and phosphate (1246.5 shift to 1256.8 cm^−1^, 1038.9 shift to 1049.1 cm^−1^, and 1158.1 shift to 1161.5 cm^−1^) towards high wavenumbers after inoculation may reflect increased metabolic activity in the host compared with susceptible cultivar Muchmore in which peaks (1423.5 and 1052.5 cm^−1^ are shifted to 1409.9 and 1049.1 cm^−1^, respectively) of cellulose were shifted towards lower wavenumber. This increased metabolic activity in Sumai3 is probably linked with the formation of defense compounds, such as those involved in the reinforcement of the cell walls. In all cultivars, the amide II peaks at 1546–1566 cm^−1^ disappeared in the presence of the fungus. The α-helix structure of amide I located around 1655 cm^−1^ in the controls have changed in diseased plants to β-sheet (1634–37 cm^−1^), indicating a change in proteins that may be used by the fungus for feeding for its survival. At the same time, no other difference was observed between healthy florets of three germplasms, except the peak located around 1540–1570 cm^−1^ which was more intense for Sumai3 (about 1566.5 cm^−1^). In the fingerprint spectral region for carbohydrate groups (1000–800 cm^−1^), no changes were detected following pathogenic infection or between healthy florets of three cultivars.

At 10 DAI, an important shift in amide I peak was observed only for both Sumai3 (1631.2 to 1638 cm^−1^) and Muchmore (1631.2 to 1659 cm^−1^) (Figure 5B). As the disease progressed from 4 to 10 DAI, the amide I peak remained same in Sumai3 and it shifted (1634. 6 to 1631.2 cm^−1^) in FL62R1 and Muchmore (1637. 9 to 1658.4 cm^−1^). Similarly, an important shift for cellulose peak in Muchmore (1406.6 to 1423.5 cm^−1^), Sumai3 (1403.1 to 1420.1 cm^−1^), FL62R1 (1413.3 to 1406.5 cm^−1^) was observed in infected floret. Also a shift of carbohydrate peak from 1055.9 to 1038.9 cm^−1^ was observed in Muchmore while a slight shift towards high wavenumber was observed in FL62R1 for the same peak.

#### Biochemical changes in rachis

Figure 5C shows the FTIR spectra of a sample composed of infected and healthy rachis of wheat cultivars examined 4 DAI. The spectra have a trend similar to that of wheat floret characterized by the same absorption peaks as described above. The broad peak at about 3390–3407 cm^−1^ is due to the stretching vibration of OH functional groups of water, alcohols, and phenols. The peak located at about 3002–3020 cm^−1^ is attributed to C-H and the doublet at about 2928–2850 cm^−1^ is attributed to asymmetric stretching modes of the CH_2_ methylene group, the common plant product. At 4 DAI, slight biochemical changes were observed between resistant, moderately resistant, and susceptible cultivars. The most important changes were: the disappearance of amide II peak at about 1550 cm^−1^ in both cultivars, Muchmore and FL62R1 after pathogenic infection; the shift of amide II peak in Sumai3 (1566.5 to 1559.7 cm^−1^); the shift of CH_2_ symmetric bending peak from 1426 to 1423 cm^−1^ in Muchmore; and the shift of amide III peak (1328.2 to 1331.6 cm^−1^) in both Sumai3 and Muchmore. The peak at 1249.9 cm^−1^ (linked to PO^−2^ asymmetric phosphate vibration) shifted to higher wavenumber (1260.2 cm^−1^) only in Sumai3 followed by FL62R1 (1249.9 to 1246.5 cm^−1^) and no change was observed in Muchmore following fungus infection.

At 10 DAI, the peak intensity of amide II (1563.1 cm^−1^) was persistent in resistant cultivar Sumai3 even after pathogenic infection as observed at 4 DAI (Figure 5D). The amide III was shifted in Muchmore from 1331 cm^−1^ (4 DAI) to 1321.4 (10 DAI). Other peaks were still persistent, even after 10 DAI in Sumai3 and other cultivars. An important difference after infection was the appearance of a peak at 1546.1 cm^−1^ for both FL62R1 and Muchmore, and the peak at 1192.1 cm^−1^ only in FL62R1.

#### Principal component analysis (PCA) of components in floret and rachis

In all cases and independent the length of after inoculation periods, PCA revealed a marked impact of FHB on floret and rachis of wheat cultivars and distinguished two clusters between infected and non-infected floret and rachis of each wheat cultivar. In most cases, PC1 explained more variation between both clusters for each wheat cultivar (data not shown); suggesting that PCA coupled with infrared spectroscopy is able to discriminate between infected and non-infected samples at an early stage of the development of pathogen infection. As demonstrated previously, the important impact of FHB on floret and rachis of wheat cultivars was observed in the IR spectra ranging from 1800 to 800 cm^−1^. Therefore, PCA was done in the spectral range from 1800 to 800 cm^−1^ to discriminate cell wall compounds between cultivars and between infected or non-infected wheat with FHB, independent of timing of inoculation for both floret and rachis.

#### Discrimination of components in wheat florets

**Figure 6:**
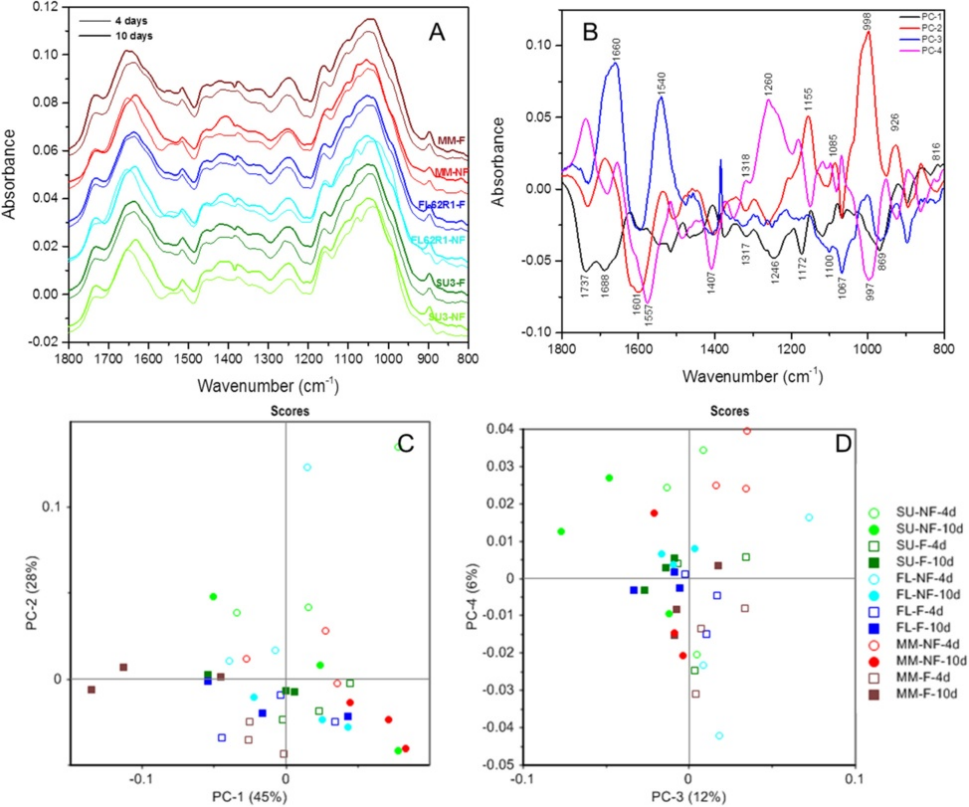
**The PCA of the FTIR spectra in the 1800–800 cm** ^**−1**^ **region (A) of the florets of wheat cultivars Sumai3, FL62R1, and Muchmore in two experimental conditions (i.e. in the presence or absence of Fusarium head blight).** Loadings plot (PC1, PC2, PC3, and PC4) of the florets of wheat cultivars using FTIR spectra **(B)**. Each point of the plot is the projection of a spectrum in the principal components PC1– PC2 space **(C)** and PC3-PC4 **(D)**. Empty symbols (○ = non-inoculated with FHB, and □ = inoculated with FHB) represent the spectra from 4 DAI and filled ones for 10 DAI (● = non-inoculated with FHB), and ■ = inoculated with FHB). The other colors are used for the different cultivars: Sumai3 (light green to dark green), FL62R1 (light blue to dark blue), and (red to brown). The percentages within the parenthesis represent the proportion of variance represented by the principal components. NF: non-infected; F: infected with Fusarium.

Spectra from control and inoculated floret of Sumai3, FL62R1, and Muchmore after inoculation periods of 4 and 10 days, were compared using PCA (Figure 6). The negative peak at around 1384 cm^−1^ in the spectra is due to the variation in the thickness of KBr pellets made as KBr has strong absorption peak at this wavenumber (Figure 6A). The total sample variation (73%) in wheat floret was explained by principal components 1 and 2 (Figure 6C). The score scatter plot of PC1 vs. PC2 indicates that the infected florets are grouped along the PC2 axis and scattered along the PC1 axis. The scores of both infected and non-infected Sumai3, and non-infected Muchmore are grouped along the positive side of PC1 whereas those of non-infected FL62R1 are spread along positive and negative sides, suggesting important differences between these three wheat germplasms. Independent of inoculation periods, score plot shows that both infected florets of FL62R1 and Muchmore are grouped in the negative side of PC2 and PC1, and significantly different from those of Sumai3. This suggests that both susceptible cultivars are affected by FHB more than the resistant cultivar, Sumai3. PC1 loadings indicated that positive influence on floret scores had values which could be assigned to pectin (around 920, 855.4, and 816.8 cm^−1^). The negative impact had values indicating pectin (1737.8, 1267.2, 969.2 cm^−1^), amide I (1688.6 cm^−1^), amide II (1586.8 and 1544.9 cm^−1^), cellulose (1516, 1463.9, 1374.2, 1317.3, 1172.7, 1117.7, 1036.7, and 896.9 cm^−1^), and xyloglucan (1066.6 cm^−1^). These negative scores suggest that changes in infected cultivars of FL62R1 tend to be located in cell wall and polysaccharides groups which were negatively affected by the presence of the fungus. PC2 explained a variability of 28%, which positively differentiated the non-infected floret at 4 days from that at 10 days, and the majority of infected florets with FHB. The loadings in the case of PC2 showed positive values for amide I (1785.1, 1686.7 cm^−1^), cellulose (1459.1, 1207.4, 1155.3, and 998.1 cm^−1^), xyloglucan (1085.9 cm^−1^) (pectin ring and xyloglucan), and pectin (926.8 and 861.2 cm^−1^). The negative PC2 loading underlines eleven peaks for hemicellulosic and cellulosic polysaccharides, and pectin (1373, 1319.3, 1601.8, 1508.3, 1473.6, 1451.4, 1401.2, 1260.4, 1068.5, 894.9, 819.7 cm^−1^). PC3 explained a variability of 12% on scores of wheat floret and separated scores into two clusters of 4 DAI in positive side and 10 DAI in negative side. The PC3 loading indicates that most of positive biochemical changes were found in pectin (1772.5, 1751.3, and 1596 cm^−1^) and amide I (1660.6 cm^−1^) whereas negative changes were located for cellulose (1475.5, 1461, 1406, 1358.8, 1180.4, 1167.8, and 896.9 cm^−1^), xyloglucan (1345.3, 1100.3, and 1067.6 cm^−1^), pectin (1326.0, 1292.3, 1271.9, 1249.8, 966.3, 856.4, 843.8, and 822.6 cm^−1^). The PC4 explained only 4% of variability and differentiated the non-inoculated samples and inoculated Sumai3 at 4 and 10 DAI in positive side. The infected FL62R1 and Muchmore were regrouped in negative side of PC4. The positive influence had peaks for pectin (1737.8, 951.8 and 825.5 cm^−1^), amide I (1654.9 cm^−1^), cellulose (1318.3, 1182.3, 1117.7, 1097.4, 1068.5, and 893 cm^*−*1^), whereas the peaks that implied negative influence were amide I (1680.8 cm^−1^), amide II (1575.7 cm^−1^), cellulose and hemicellulose (1485.1, 1440.8, 997.2), and pectin (1150.5, 1082.0, 923.9, and 862.1 cm^−1^).

#### Discrimination of components in the wheat rachis

**Figure 7:**
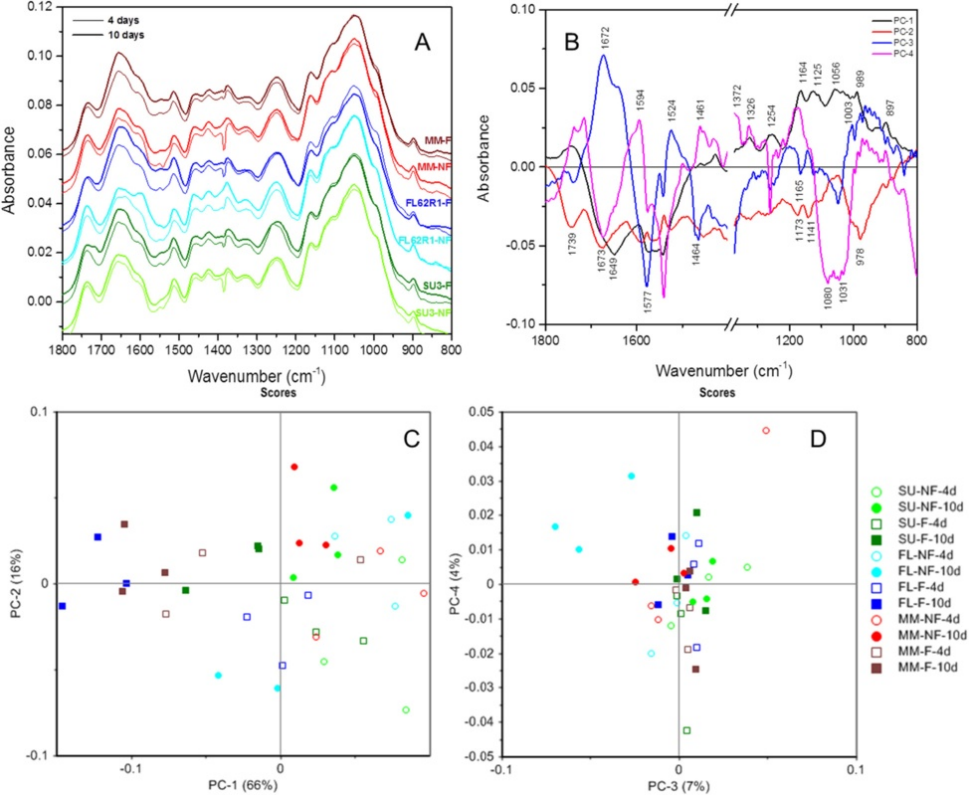
**The PCA of the FTIR spectra in the 1800–800 cm** ^**−1**^ **region (A) of the rachises of wheat cultivars Sumai3, FL62R1, and Muchmore in two experimental conditions (i.e. in the presence or absence of Fusarium head blight).** Loadings plots (PC1, PC2, PC3, and PC4) of the rachises of wheat cultivars using FTIR spectra **(B)**. Each point of the plot is the projection of a spectrum in the principal components PC1– PC2 space **(C)** and PC3-PC4 **(D)**. Empty symbols (○ = non-inoculated with FHB, and □ = inoculated with FHB) represent the spectra from 4 DAI and filled ones for 10 DAI (● = non-inoculated with FHB), and ■ = inoculated with FHB). The other colors are used for the different cultivars: Sumai3 (light green to dark green), FL62R1 (light blue to dark blue), and (red to brown). The percentages within the parenthesis represent the proportion of variance represented by the principal components. NF: non-infected; F: infected with Fusarium.

The total sample variation (82%) in the rachis of the wheat cultivars was explained by principal components 1 and 2 (Figure 7). The PC1 (66%) clearly distinguish infected rachis of Muchmore (4 and 10 DAI) and the infected rachis of FL62R1 and Sumai3 (10 DAI) in separate clusters in the negative side of PC1 whereas non-infected rachis and infected rachis of FL62R1 and Sumai3 (4dai) are scattered in the positive side of PC1. PC1 loadings indicated that positive influence of FHB on rachis scores had values for pectin (1785, 1742.6, 1326.0, 930.6, 920, 870.8, 838.99 and 821.6 cm^−1^), cellulose and hemicellulose (1475.5, 1460.1, 1428.2, 1365.5, 1254.6, 1164.9, 1125.4, 1110.9, 1056.9, 1033.8, 1019.3, 1002.9, 989.4, and 897.8 cm^−1^). PC2 explained a variability of 16% and the loadings in the case of PC2 showed positive values for pectin (850.6, 837.1, and 824.5 cm^−1^). The most important peaks in the negative PC2 loading were for amide I (1673.2 cm^−1^), amide II (1562.3, 1556.5, and 1524.7 cm^−1^), cellulose, hemicellulose and pectin groups (1739.71493.8, 1453.3, 1423.4, 1395.4, 1375.2, 1334.7, 1323.1, 1288.4, 1276.8, 1265.2, 1173.6, 1141.8, 1109, 1059.8, 978.8, 897.8, and 873.7 cm^−1^). PC3 and PC4 explained a variability of 11% on scores of the rachis of wheat and showed two clusters. In the case of PC3, a positive impact on scores corresponds to wavenumbers for (1672.2 cm^−1^), amide II (1524.6 cm^−1^) and cell wall polysaccharides (1209.3, 1187.1, 1143.7, 1003.9, 977.9, 963.4, 949.9, 934.5, 921.9, 893.0, 864.1, and 816.8 cm^−1^). The negative impact on the scores had wavenumbers indicating phenolic regions of lignin and pectin (1793.7, 1779.3, 1768.6, and 1738.7 cm^−1^), amide II (1577.7 and 1542.0 cm^−1^), and cellulose, hemicellulose, and pectin (1464.9, 1433.0, 1413.8, 1337.6, 1305.8, 1294.2, 1280.7 and 1248.9, 1165.9, 1123.5, 1112.9, 1089.7, 1048.3, and 840.9 cm^−1^). The PC4 explained only 4% of variability of the impact of FHB on the rachis of the wheat cultivars. The PC4 loading obtained for every three sets of variable (Sumai3, FL62R1 and Muchmore) had significant positive value corresponding to pectin and phenolic groups (1786.9, 1736.8, and 1714.6 cm^−1^), amide II (1594.1and 1569.9 cm^−1^) cellulose and hemicellulose (500.6, 1461.9, 1372.3, , 1168.8, 1135.1, 999.1, and 986.5, 897.8 cm^−1^) and pectin (1327.9, 943.0, 873.7, and 840.9 cm^−1^). Negative influence had wavenumbers around amide I (1674.1 cm^−1^), amide II (1575.8 and 1540.1 cm^−1^) and polysaccharides (1478.4, 1440.8, 1410.9, 1349.1, 1338.6, 1304.8, 1294.2, 1261.4, 1235.4, 1214.1, 1089.7, 1082.1, 1056.9, 1024.2, and 1021.3 cm^−1^).

## Discussion

To our knowledge, this paper is the first combining PCI and FTIR spectroscopy to report on the defense mechanism of wheat against FHB. In the current study, we have presented a new approach for evaluating the resistance mechanisms of different lines to FHB based on the use of synchrotron based imaging techniques and FTIR spectroscopy in comparing resistance and susceptible wheat cultivars. The use of PCI coupled with FTIR provides a new approach to the study of structural and chemical responses of the host to FHB infection, traditionally analyzed by microscopy using histochemical methods.

Resistance to FHB varies greatly among wheat genotypes. The Chinese cultivar Sumai3 is the best known and most widely documented resistant cultivar [50]. Sumai3 pedigree contains the two moderately susceptible cultivars Funo and Taiwanmai [51]. It has been rated as resistant to highly resistant in many screening experiments. Although Sumai3 possesses very good type II FHB resistance, it is susceptible to other diseases and seeds shatter easily. Here, we have compared the infected and non-infected floret and rachis of Sumai3 to those of tolerant cultivar, FL62FR1 and susceptible cultivar, Muchmore. Results demonstrate significant differences in mass densities between healthy floret and infected floret with FHB at 4 DAI. Healthy florets appear in white colors and filled with internal structures while infected ones are largely empty and transparent suggesting that PCI could be a useful tool in plant disease diagnosis. Several researchers underlined the use of PCI as a non-destructive imaging technique in plant systems for studying physiology of plants. Examples of such PCI studies include: cavitation and water refilling processes [52], plant tissues in 3D [53], X-ray imaging of leaf venation [54] or studying mutualistic association of plants to arbuscular mycorrhizal fungi [55]. Similarly, Kim and Joon Lee [56] presented PCI as a minimally invasive method to observe anatomical structures and sap flow dynamics in rice xylem. Overall, our synchrotron based PCI results have highlighted significant differences between infected and non-infected florets. However it was difficult to precisely characterize real difference between non-inoculated resistant and susceptible cultivars in term of floret structures due to the complexity of the internal structures. Finally, a total loss of cell viability was observed in infected florets of the Muchmore cultivar.

The PCI revealed significant differences between infected and non-infected rachis of different wheat cultivars. Without infection by FHB, the form (internodes) and thickness of edge of the rachis and the cavitation inside the rachis were different from one cultivar to another. In the presence of the fungus, the PCI shows that structures could be lost or altered, notably in susceptible cultivar Muchmore in which the structure of rachis has been totally altered and became more transparent to X-rays as a sign of infection compared to the control rachis. The cavitation region, visible as a transparent area in phase contrast images, in which water movement occurs in the rachis, became thinner in FHB infected samples. We hypothesize that the bright structure located in the internodes of Sumai3 rachis (Figure 4) and the presence of more cavitation regions with xylem and phloem cell-wall structures contributes to the resistance of Sumai3 to FHB. These characteristic structures are believed to limit the growth and the spread of the fungal mycelium through water flow within the rachis which is considered to be the causes of spike blight symptoms [57,58]. These researchers found that anatomical features associated with type II resistance include smaller diameter vessels, denser vascular bundles in the rachis, strong thickened cortical sclerenchyma and cell walls, and short internodes in the upper part of the rachis. These observations are partially corroborated by our X-ray imaging. Accordingly, 3D PCI may be ideal to reveal the internal structures of the rachis and could be an appropriate tool for fast screening of the resistant cultivars against scab blight.

Testing thousands of lines in a breeding program each year may not be feasible for routine selection of resistant lines to FHB. Therefore, selection based on other related characters such as FHB severity or percent scabby seed has been proposed [7,59]. However, the identification and evaluation of asymptomatic infection in wheat spikelets via light and electron and confocal microscopy is time consuming and costly [60]. In the current study, we proposed PCA of FTIR spectroscopy of bulk florets and rachises as a new fast technique in comparing resistant line Sumai3, tolerant line FL62R1, and susceptible line Muchmore of wheat to FHB. Independent of the length of post-inoculation periods, PCA results showed significant differences between infected and non-infected florets of wheat cultivars. In the resistant cultivar Sumai3, the shifts in spectral peaks after inoculation were high (1375.9 to1382.7 cm^−1^, 1246.5 to1256.8 cm^−1^, 1038.9 to 1049.1 cm^−1^, and 1158.1 to 1161.5 cm^−1^). In Muchmore, the peaks at 1423.5, 1375.9, and 1052.5 cm^−1^ were shifted to 1409.9, 1379.2, and 1049.1 cm^−1^ respectively. For FL62R1 and other cultivars the disappearance of amide I α-helix peak (1546–66 cm^−1^) and the change of amide I (1655 cm^−1^) in control to β-sheet (1634–37 cm^−1^) in diseased plant when coupled with a change in the cellulose peak (1049.1 cm^−1^) indicates that both amide I and cellulose peaks may be used as a marker of pathogenic infection with FHB. The only difference observed between the spectra of florets among the different cultivars is the peak around 1540–70 which was more intense for Sumai3 (~1566.5 cm^−1^), suggesting that peak could be used as resistance marker for FHB. Overall, the analysis of spectra of infected and non-infected florets supports a conclusion of increased metabolic activity in the host. The most important peaks representing amide I, amide II, cellulose, hemicellulose, and pectin [45,46,61,62] may be linked with the formation of defense compounds, such as those involved in the reinforcement of the cell walls. Additionally, the timing after infection had also influenced significantly the biochemical changes in the florets of wheat cultivars infected with or without FHB infection. For example, there was an important shift in 1631 cm^−1^ and 1406.6 cm^−1^ observed in Muchmore (to 1659 and 1420.1 cm^−1^) and Sumai3 (to 1638 and 1420.1 cm^−1^). In the carbohydrates region, a significant decrease of peak from 1055.9 to 1038.9 cm^−1^ was recorded only for Muchmore. The PCA has explained more than 73% of total variability principally due to the *Fusarium* infection. PCA showed that infected florets are grouped along PC2 axis and scattered along PC1 axis. The florets from Sumai3 appeared genetically different from those from other cultivars, Muchmore and FL62R1 in terms of response to FHB infection. The positive loading of PC1 was prominent in the spectra linked to carbohydrates 920, 855 and 816.8 cm^−1^ which assigned to pectin and xyoglucan while the negative loadings is dominated by peaks belongings to pectin (1737.8, 1267.2, and 969.2 cm^−1^), proteins (1688.6 and 1544.9 cm^−1^), cellulose vibrations (1516, 1463.9, 1374.2, 1313.3, 117.7, and 896.9 cm^−1^), and hemicellulose (1066.6 cm^−1^), which may play an important role in host defense against the invasion by FHB and may be useful for fast routine screening methods of highly resistant cultivars against this devastating pathogenic fungus.

The spectrum of Fusarium has been largely studied and contains a very broad band in the region of 3650–3000 cm^−1^ with a peak near 3260 cm^−1^. The amide I and II bands (1700–1485 cm^−1^), which likely arise from fungal proteins, were prominent. The fingerprint region of FHB hyphae was dominated by a broad peak near 1035 cm^−1^ [44,63]. Accordingly, the broad and strong absorption of this peak in the floret of infected cultivars in combination with a visible alteration in the protein region (α-helix turned to β-sheet) following pathogenic infection, maybe used as a signature marker for pathogenic infection with FHB. This corroborates the findings of Barran et al. [64] who reported that the cell walls of a FHB hyphae contained 66% carbohydrate, 7.3% proteins, 5.5% lipid, and 1.8% ash. In agreement with the published literature, PCA applied to FTIR spectra underlines a substantial role of cell wall compounds in reaction to FHB as demonstrated by the relatively higher spectral peaks in Sumai3 after inoculation with FHB (1382.7, 1256, 1049.1 cm^−1^), which may reflect increased metabolic activity linked with the formation of defense compounds such as those involved in the reinforcement of cell walls as shown previously by Martin et al. [33] on Dutch elm disease.

Measurement of the spread of FHB within a spike has been recognized as a relatively reliable index of cultivar resistance [65,66]. The spread of the disease within a spike was characterized by two distinct stages; spread into rachis and through the rachis into other florets via rachis internodes [7]. These stages are mostly affected by resistance genes cultivars [7]. The structure of the rachis was known to display a significant role in the resistance. Consequently, understanding the impact of FHB on chemical structure of the rachis in resistant and susceptible cultivars could help develop new selection strategies against this devastating pathogen. FTIR spectra have shown the same absorption peaks as described above for the wheat florets and slight difference was recorded between resistant, moderately resistant, and susceptible cultivars. The peak 1550 cm^−1^ related to lignin vibration disappeared in both cultivars, Muchmore and FL62R1 after pathogenic infection at 4 DAI and is still predominant in Sumai3, even after 10 DAI, suggesting the implication of lignin in resistance II of Sumai3 against FHB**.** We noticed a shift of peak related to cellulose CH_2_ symmetric bending at about 1426 to 1423 cm^−1^ in Muchmore, and the shift of peak at about 1328.2 to 1331.6 cm^−1^ in both Sumai3 and Muchmore. The peak 1249.9 cm^−1^ is linked to PO^−2^ asymmetric phosphate vibration was increased only in Sumai3 following fungus infection. This indicates a potential role in resistance of Sumai3 against this pathogenic fungus. It was known that phosphorylated proteins are key elemental function in the activation of phenylpropanoids biosynthetic genes involved in the elaboration of lignin precursors, phytoalexins and the secondary signal salicylic acid as early responses to pathogen invasion [67-69]. Therefore, the absorption peak at 1530–1563 cm^−1^ (amide II, lignin), which was persistent in the rachis of Sumai3 and coupled with increased shift in absorption peaks of 1323 cm^−1^ and 1245 cm^−1^ in Sumai3 inoculated with FHB may be considered as a marker of resistance against this pathogenic fungus. These conclusions could be of significance in developing early screening methods based on FTIR in selecting cultivars resistant against FHB and avoiding time consuming and costly screening techniques. By comparing both inoculation periods, the most important changes were a shift of peak 1331 to 1321.4 cm^−1^ in the case of Muchmore and the appearance of peak 1546 cm^−1^ for both cultivars Muchmore and FL62R1 as a reaction to pathogenic infection. The peak 1192.08 cm^−1^ appeared only in FL62R1 and may related to the host immunity system response to the presence of pathogenic fungus.

The PCA of FTIR spectra underlined significant differences in rachis of the wheat cultivars and between those infected and non-infected by FHB. The total sample variation (82%) in the rachis of the wheat cultivars was explained by principal components 1 and 2. PC1 (66%) clearly distinguish infected rachis of Muchmore (4 and 10 DAI) and the infected rachis of FL62R1 and Sumai3 (10 DAI) in separate clusters in the negative side of PC1 whereas non-infected rachis and infected rachis of FL62R1 and Sumai3 (4dai) are scattered in the positive side of PC1. The positive influence of PC1 on rachis scores had values around 1785, 1742.6, and 1326.0, 1254.6 cm^−1^ (alkyl ester, pectin, lignin and phenolic vibrations), 1475.5, 1460.1, 1428.2, and 1365.5 cm^−1^ (cellulose and lignin vibration), and polysaccharides regions including some wavenumbers denoting hemicellulose and pectin (1164.9, 1125.4, 1110.9, 1056.9, 1033.8, 1019.3, 1002.9, 989.4, 930.6, 920, 897.8, 870.8, 838.99 and 821.6 cm^−1^). PC2 explained a variability of 16% and the loadings in the case of PC2 showed positive values for pectin and polysaccharides (850.6, 837.1, 824.5 cm^−1^). The most important peaks in the negative PC2 loading were dominated by carbonyl compounds such as phenolics and lignin (1739.7 cm^−1^), proteins (1673.2, 1562.3, 1556.5 and 1524.7), celluloses and pectin (1493.8, 1453.3, 1423.4, 1395.4, 1375.2, 1334.7, 1323.1, 1288.4, 1276.8, and 1265.2 cm^−1^), and polysaccharides (1173.6, 1141.8, 1109, 1059.8, 978.8, 897.8, and 873.7 cm^−1^). These results highlighted that pathogenic infection induced host resistance in three cultivars are represented by proteins and cell wall compounds. Our results are in line with those of Peiris et al. [44] who reported a shift in the peak position of 1035 cm^−1^ along with increased absorptions at 1160, 1203, 1313, and 1375 cm^−1^, likely due to the influence either by FHB hyphae directly or by another metabolic change in infected tissue resulting from fungal invasion.

The FTIR spectroscopy has been shown to be a powerful attractive technique for the study of biological macromolecules and of complex biological systems such as tissues and cells [70,71]. The highlighted and prominent peaks in this study are largely linked to cell-wall compounds, carbonyl esters and polysaccharides in explaining variations between exanimated wheat cultivars with or without fungal infection. The PCI and FTIR results support the use of these techniques for screening for relative resistances in wheat to FHB. The sections imaged with PCI in internodes of the rachis of Sumai3 may act as barriers to pathogen growth or diffusion of toxins, allowing the formation of new healthy xylem [72] and may linked to lignification defense mechanisms of plant as supported by the persistence of the absorption peak for amide II and lignin (1530–1563 cm^−1^) with an increased peak shift denoting for cellulose and hemicellulose (1323 cm^−1^ and 1245 cm^−1^) following pathogenic infection.

## Conclusions

Marker-assisted selection offers many advantages to breeders by reducing the time necessary for testing the plant materials, and the effort and money required to accomplish a successful gene introgression in the genotype of interest. In the current study we examined the potential of synchrotron based phase contrast imaging in combination with Principal Component Analysis applied to FTIR spectra for an easy and rapid comparison of structural and molecular changes in the host following pathogenic infection to determine the resistance mechanisms. The PC images show significant differences between infected and non-infected florets, however, no pronounced difference between non-inoculated resistant and susceptible cultivar in term of floret structures could be determined due to the complexity of the internal structures. In the rachises, PC images revealed significant differences between infected and non-infected rachis of different wheat cultivars. In addition, the results obtained in this current study provide an unique, consistent, and significant spectral biomarkers for infected region with FHB and specific spectral biomarkers for selecting resistance cultivars against FHB based on data comparison of highly resistant cultivar Sumai3 and highly susceptible cultivar Muchmore. Using these biomarkers, it is possible to discriminate between the different examined wheat cultivars. Additionally, the fact that the final results could be obtained from the infected spikelet’s during a very short time from a small amount of sample and by a simple procedure, support the possibility of developing FTIR spectroscopy as a reliable method for rapid identification and discrimination between infected and non-infected plants with FHB and between different wheat cultivars in terms of resistance against FHB. This technique would be very useful as an additional tool to molecular methods largely used in plant biology and microbiology for studying plant-microbe interaction such as RNA-Seq and Proteomics.

## Methods

### Fungal material and inoculum preparation

Wild-type *Fusarium gramineraum* (isolate DAOM 180379 from the Canadian collection of fungal cultures, Ottawa, Ont.), which was transformed to constitutively express Green Fluorescent Protein (GFP) in both macroconidia and hyphae was used in this study. Fresh inoculum was taken from the stored type culture at monthly intervals. For the production of macroconidia, a plug of actively growing *Fg*-GFP (Green Fluorescent Protein *Fusarium gramineraum*) was placed in the center of a petri dish containing Soft Nutrient Agar (SNA). Plates were placed under a combination of fluorescent and UV lights for 5 days at 23°C. Macroconidia were harvested by pouring a small amount of sterile water over the culture in the petri dish and then by either gently scraping the surface with a bent glass pipette or washing with a gentle stream of water, using a pipette. A working concentration of approximately 2500 macroconidia/mL was attained by concentrating the suspension or by dilution with sterile water as required. A concentration of 10^5^ macroconidia/ mL was used for inoculation.

### Plant material and infection procedure

All experiments were conducted in the environment-controlled growth chamber due to restrictions on inoculating with a transformed fungus in the field. Canadian germplasm ‘FL62R1’ (or FL) was developed by Drs. Andre Comeau and Francois Langevin (AAFC- Québec) [73]. Canadian germplasm ‘Muchmore’ (or MM) was developed by Drs. Ron DePauw and Richard Cuthbert (AAFC-Swift Current) [74]. Seeds of resistant ‘Sumai3’, Canadian germplasm ‘FL62R1’, and ‘Muchmore’ were sown in peat pots (diameter, 12.7 cm) and maintained in a growth chamber at 20°C: 16°C day: night cycle, with 16 h of light per day until flowering. Pots were watered by hand at the base of the plants. At mid-anthesis, single floret inoculation with *Fg* strain was carried out by pipetting 10 μl of the macroconidia suspension (10^5^ per ml) between palea and lemma. For disease severity test, four florets of two spikelets in the middle of head were inoculated per each genotype. For other experiments, 10 spikelets midway along the spike were point inoculated. Inoculated plants were incubated in a dew chamber for 2 days and then moved back to growth chamber for the rest of the experiment.

Diseased spikelets were expressed as means ± standard error. Data were analyzed by using two-way ANOVA test of the statistical analysis system (SAS Institute, version 9.1, Cary, NC, USA). Mean values were compared using Fisher’s LSD test at statistical significance P = 0.01.

Microscopy was performed on fresh and hand sectioned material, using fluorescence stereomicroscope system (SteREO Lumar.V12, Zeiss). Photos were taken with Zeiss AxioCam HR colored camera.

### Synchrotron based phase contrast X-ray imaging

Fresh spikes were excised from the plants ~ 4 hours before X-ray imaging and the cut spikes were stored in a plastic bag at room temperature. X-ray images of wheat spikes were recorded using the phase contrast imaging technique at the Biomedical and Imaging Therapy (BMIT) beamline at the Canadian Light Source. The X-ray energy was selected to be 18 keV, the lowest possible in the beamline and a 0.5 mm thick aluminum filter was used before the monochromator to reduce the heat load on the monochromator. An 8.75 μm resolution detector was used and most images were recorded in less than a second exposure time. Two dimensional transmission images (projection images) were collected and the projection images were corrected for the dark signal from the detector (dark signal correction) and flat signal (flat-field correction) for imperfections from the monochromator and scintillator screens. The dark and flat images were collected at the beginning of the imaging session for each spikelet. The spike was kept inside a 18 mm diameter falcon tube during imaging to prevent any movement of the spike when collecting data along the length of the spikelets.

### FTIR spectroscopy

All FTIR spectroscopy were collected at the mid infrared beamline (01B1-1) at the Canadian Light Source Inc., Saskatoon, Canada using the glowbar source (silicon carbide) as the infrared source. The Bruker - IFS 66 V/S spectrophotometer (Bruker Optics, Ettlingen, Germany) with a Deuterated triglycine sulphate (DTGS) detector was used for the FTIR measurements.

The floret and rachis samples of infected and non-infected spikelets were prepared by the method described by Naumann *et al*. [75]. Floret and rachis samples were first dried using a freeze drier and ground to a fine powder. About 1 mg of freeze dried and powdered sample was homogenized with about 2.0 mg of dry potassium bromide (KBr) using pestle and mortar and made into a pellet. Transmission infrared spectrum was obtained from the finally prepared pellet for replicate samples. Each IR spectrum was recorded in the mid infrared range of 4000–800 cm^−1^ wavenumbers at a spectral resolution of 2 cm^−1^. Each sample spectrum is an average of 64 scans and pure KBr spectra (average of 512 scans) was recorded for normalizing all sample spectra. The normalized spectra were baseline corrected using the rubber band correction (64 points) and vector normalized using the OPUS software (version 7.0, Bruker Optics Inc., Billerica, MA). All FTIR spectra shown here are the average spectra from three replicates. The FTIR peaks cited in the supplemental tables were determined using the Quick Peaks routine in OriginPro with the settings of local maximum at 0% threshold height, no baseline, and area at Y = 0.

### Principal component analysis

Principal Component Analysis (PCA) is one of the most common methods used in IR spectroscopy to look at the spread of the data. By performing a PCA the variance-covariance structure of p variables through k linear combinations, called Principal Components (PC), can be explained. The purpose of PCA is to reduce the number of variables and provide an easy graphical representation on the spread of the data [45]. Geometrically, a PCA is a vector space transform. It can be seen as a transformation applied to the coordinate axis rather than to the data set. The new coordinates are the principal components such that the first PC represents the direction of greatest variability, the second greatest variance lies on the second PC and so on. This method is especially useful in the interpretation of FTIR spectra, which show peak diversity and complication depending on the source of the sample. The Unscrambler 10.1 (Camo Software AS., Norway) was used for performing PCA. Each wavelength of FTIR was treated as an equally weighted variable in this analysis.

### Supporting data

The data sets supporting the results of this article are included within the article and its additional files.

## Additional files

Additional file 1: Figure S1. Infection progress of *Fg*-GFP in wheat rachis at 10 DAI. A schematic illustration of wheat spike is shown in left side. For simplicity only one floret per spikelet is shown. Thick yellow arrow indicates the inoculation site. Red lines indicate the approximate position of hand sections. RI, Rachis internode; RA, Rachilla. Each sample was taken two photos under epiflurousent and white light microscope. Additional file 2: Tables S1–S4. Assignments of FTIR absorption peaks (cm^−1^) of wheat florets at 4 days after inoculation **(Table S1).** Assignments of FTIR absorption peaks (cm^−1^) of wheat florets at 10 days after inoculation **(Table S2).** Assignments of FTIR absorption peaks (cm^−1^) of wheat rachis at 4 days after inoculation **(Table S3).** Assignments of FTIR absorption peaks (cm^−1^) of wheat rachis at 10 days after inoculation **(Table S4).**

## References

[1] Jansen C, Von Wettstein D, Schafer W, Kogel KH, Felk A, Maier FJ (2005). Infection patterns in barley and wheat spikes inoculated with wild-type and trichodiene synthase gene disrupted *Fusarium graminearum*. PNAS.

[2] Liu X, Tang WH, Zhao XM, Chen L (2010). A network approach to predict pathogenic genes for *Fusarium graminearum*. PloS One.

[3] Jonkers W, Dong Y, Broz K, Kistler HC (2012). The Wor1-like protein FGP1 regulates pathogenicity, toxin synthesis and reproduction in the phytopathogenic fungus Fusarium graminearum. Plos Pathog.

[4] Gilbert J, Tekauz A (2000). “Review: Recent developments in research on fusarium head blight of wheat in Canada. Can J Plant Pathol.

[5] Desjardins AE, Hohn TM (1997). Mycotoxins in plant pathogenesis. MPMI.

[6] Goswami RS, Kistler HC (2004). Heading for disaster: *Fusarium graminearum* on cereal crops. Mol Plant Pathol.

[7] Bai GH, Shaner G (1994). Scab of wheat: prospects for control. Plant Dis.

[8] McMullen M, Jones R, Gallenberg D (1997). Scab of wheat and barley: A re-emerging disease of devastating impact. Plant Dis.

[9] Bushnell WR, Hazen B, Pritsch C, Leonard KJ, Bushnell WR (2003). Histology and physiology of Fusarium head blight. Fusarium Head Blight of Wheat and barley.

[10] Van Sanford D, Anderson J, Campbell K, Costa J, Cregan P, Griffey C, Hayes P, Ward R (2001). Discovery and deployment of molecular markers linked to FHB resistance: An integrated system for wheat and barley. Crop Sci.

[11] Mesterházy Á, Bartók T, Lamper C (2003). Influence of cultivar resistance, epidemic severity, and Fusarium species on the efficacy of fungicide control of Fusarium head blight in wheat and deoxynivalenol (DON) contamination of grain. Plant Dis.

[12] Henriksen and Elen (2005). Natural Fusarium Grain Infection Level in Wheat, Barley and Oat after Early Application of Fungicides and Herbicides. J Phytopathol.

[13] Miedaner T (1997). Breeding wheat and rye for resistance to Fusarium diseases. Plant Breed.

[14] Gaurilcikiene I, Mankeviciene A, Suproniene S (2011). The effect of fungicides on rye and triticale grain contamination with Fusarium fungi and mycotoxins. Zemdirbyste (Agriculture).

[15] Stack RW: *Return of an old problem: Fusarium head blight on small grains plant health progress – plant health reviews*, 2000. http://www.apsnet.org/publications/apsnetfeatures/Pages/headblight.aspx

[16] Pirgozliev SR, Edwards SG, Hare MC, Jenkinson P (2003). Strategies for the control of Fusarium head blight in cereals. Eur J Plant Pathol.

[17] Ramirez L, Chulze S, Magan N (2004). Impact of environmental factors on growth and deoxynivalenol production by *Fusarium graminearum* isolates from Argentinean wheat. Crop Prot.

[18] Somers DJ, Thomas J, DePauw R, Fox S, Humphreys G, Fedak G (2005). Assembling complex genotypes to resist Fusarium in wheat (*Triticum aestivum* L.). Theor Appl Genet.

[19] Ban T, Suenaga K (2000). Genetic analysis of resistance to Fusarium head blight caused by *Fusarium graminearum* in Chinese wheat cultivar Sumai3 and the Japense cultivar Saikai 165. Euphytica.

[20] Singh RP, Ma H, Rajaram S (1995). Genetic analysis of resistance to scab in spring wheat cultivar Frontana. Plant Dis.

[21] Buerstamyer H, Steiner B, Hartl L, Griesser M, Angerer N, Lengauer D (2003). Molecular mapping of QTLs for Fusarium head blight resistance in spring wheat. II. Resistance to fungal penetration and spread. Theor Appl Genet.

[22] Boenisch MJ, Schafer W (2011). Fusarium gramineaum forms mycotoxin producing infection structures on wheat. BMC Plant Biol.

[23] Miller SS, Watson EM, Lazebnik J, Gulden S, Balcerzak M, Fedak G, Ouellet T (2011). Characterization of an alien source of resistance to Fusarium head blight transferred to Chinese Spring wheat. Botany.

[24] Zuther E, Huang S, Jelenska J, Eilenberg H, Arnold EM, Su X, Sirikhachornkit A, Podkowinski J, Zilberstein A, Haselkorn R, Gornicki P (2004). Complex nested promotors control tissue-sepcific expression of acetyl-CoA carboxylase genes in wheat. PNAS.

[25] Gonzalez-Melendi P, Fernandez-Pacheco R, Coronado MJ, Corredor E, Testillano PS, Risueno MC, Marquina C, Ibarra MR, Rubiales D, Perez-De-Luque A (2008). Nanoparticles as Smart Treatment-delivery Systems in Plants: Assessment of Different Techniques of Microscopy for their Visualization in Plant Tissues. Ann Bot.

[26] Yuasa H: **On the advantage of the X-ray examination of certain classes of materials and insects subject to the plant quarantine regulations.** In: *Proceedings of the Third Pan-Pacific Science Congress*, (30 October - 11 November), Tokyo 1926:1141. (Abstract).

[27] Fenton FA, Waite WW (1932). Detecting pink bollworms in cottonseeds by the X-ray. J Agr Res.

[28] Hounsfield GN (1973). Computed transverse axial scanning (tomography). Part I. Description of system. British J Radiol.

[29] Mooney S, Pridmore T, Helliwell J, Bennett M (2012). Developing X-ray Computed Tomography to non-invasively image 3-D root systems architecture in soil. Plant Soil.

[30] Rong-Changchen DD, Mancini L, Menk R, Rigon L, Xiao TQ, Longoa R (2012). PITRE: software for phase-sensitive X-ray image processing and tomography reconstruction. J Synchrotron Radiat.

[31] Griffiths PR, de Haseth JA (1986). Fourier transform infrared spectrometry.

[32] McCann MC, Chen L, Roberts K, Kemsley EK, Sene C, Carpita NC, Stacey NJ, Wilson RH (1997). Infrared microspectroscopy: Sampling heterogeneity in plant cell wall composition and architecture. Physiol Plantar.

[33] Martin JA, Solla A, Woodwards S, Gil L (2005). Fourier transform-infrared spectroscopy as a new method for evaluating host resistance in the Dutch elm disease complex. Tree Physiol.

[34] Yee N, Benning LG, Phoenix VR, Ferris FG (2004). Characterization of metal-Cyanobacteria sorption reactions: A combined Macroscopic and infrared spectroscopic investigation. Environ Sci Technol.

[35] Freitas PAM, Iha K, Felinto MCFC, Suárez-Iha MEV (2008). Adsorption of di-2-pyridyl ketone salicyloylhydrazone on amberlite XAD-2 and XAD-7 resins: characteristics and isotherms. J CollInterface Sci.

[36] Taoutaou A, Socaciu C, Pamfil D, Fetea F, Balazs E, Botez C (2012). New markers for potato late blight resistance and susceptibility using FTIR spectroscopy. Not Bot Horti Agrobo.

[37] Erukhimovitch V, Tsror L, Hazanovsky M, Huleihel M (2010). Direct identification of Potato’s fungal phytopathogens by Fourier-transform infrared (FTIR) microscopy. Spectroscopy.

[38] Mann DGJ, Labbe N, Sykes RW, Gracom K, Kline L, Swamidoss IM, Burris JN, Davis M, Stewart CN (2009). Rapid assessment of Lignin content and structure in switchgrass (Panicum virgatum L.) grown under different environmental conditions. Bioenerg Res.

[39] Bertoluzza A, Bottura G, Lucchi P, Marchetti L, Zechini D’Aulerio A (1999). Molecular Monitoring of horse chestnut leaves affected with biotic and abiotic disorders. J Plant Pathol.

[40] Hazen S, Hawley RM, Davis G, Henrissat B, Walton JD (2003). Quantitative trait loci and comparative genomics of cereal cell wall composition. Plant Physiol.

[41] Bhuiyan N, Selvaraj G, Wei Y, King J (2009). Role of lignification in plant defense. Plant Signal Behav.

[42] Vorwerk S, Somerville S, Somerville C (2004). The role of Plant cell wall polysaccharide composition in disease resistance. Trends Plant Sci.

[43] Socha JJ, Westneat MW, Harrison JF, Waters JS, Lee WK (2007). Real-time phase-contrast x-ray imaging: a new technique for the study of animal form and function. BMC Biol.

[44] Peiris KHS, Bockus WW, Dowell FE (2012). Infrared Spectral Properties of Germ, Pericarp, and Endosperm Sections of Sound Wheat Kernels and those damaged by *Fusarium Graminearum*. Applied Spectrosc.

[45] Szymanska-Chargot M, Zdunek A (2013). Use of FTIR spectra and PCA to the bulk characterization of cell wall residues of fruits and vegetables along a fraction process. Food Biophys.

[46] Kacurakova M, Wlison RH (2001). Developments in mid-infrared FT-IR spectroscopy of selected carbohydrates. Carbohyd Polym.

[47] Kacurakova M, Capek P, Sasinkova V, Wellner N, Ebringerova A (2000). FT-IR study of plant cell wall model compounds:pectic polysaccharides and hemicelluloses. Carbohyd Polym.

[48] Fengel D, Wegener G (1989). Wood, Chemistry, ultrastructure, reactions.

[49] Dorado J, Almendros G, Field JA, Sierra-Alvarez R (2001). Infrared spectroscopy analysis of hemp (*Cannabis sativa*) after selective delignification by Bjerkandera sp. at different nitrogen levels. Enzyme Microb Technol.

[50] Basnet BR, Glover KD, Ibrahim AMH, Yen Y, Chao S (2011). QTL on chromosome 2DS of ‘Sumai3’ increases susceptibility to Fusarium head blight in wheat. Euphytica.

[51] Bai GH, Plattner R, Desjardin A, Kolb F (2001). Resistance to Fusarium head blight and deoxynivalenol accumaulated in wheat. Plant Breed.

[52] Yanling X, Tiqiao X, Guohao D, Yajun T, Huiqiang L, Biao D, Honglan X, X H: **Observation of cavitation and water-refilling processes in plants with X-ray phase contrast microscopy.** *Nucl Sci Tech* 2013, **24:**060101–6.

[53] Staedler YM, Masson D, Schonenberger J: **Plant tissues in 3D via X-ray tomography: simple contrasting methods allow high resolution imaging.** *PloS One* 2013, **8:**e75295. 10.1371/journal.pone.0075295 PMC3785515 24086499

[54] Blonder B, De Carlo F, Moore J, Rivers M, Enquist BJ (2012). X-ray imaging of leaf venation networks. New phytol.

[55] Yun W, Pratt ST, Miller RM, Cai Z, Hunter DB, Jarstfer AG, Kemner KM, Lai B, Lee HR, Legnini DG, Rodrigues W, Smith CI (1998). X-ray imaging and microspectroscopy of plants and fungi. J Synchrotron Radiat.

[56] Kim HK, Lee SJ (2010). Synchrotron X-ray imaging for nondestructive monitoring of sap flow dynamics through xylem vessel elements in rice leaves. New Phytol.

[57] Yu QL, Liu TR, Liu YX, Jia GX (1996). Pathological anatomy of the rachis in wheat varieties with resistance against scab. J Heilongjiang August First Land Reclamation Univ.

[58] Zhang YH, Ye HZ (1993). A study of histology of resistance of wheat to head scab (*Fusarium graminearum*). J Sichuan Agric Univ.

[59] Buerstamyer M, Huber K, Heckmann J, Steiner B, Nelson JC, Buerstmayr H (2012). Mapping QTL for Fusarium head blight resistance and morphological and development traits in three backcross populations derived from Triticum x triticum durum. Theor Appl Genet.

[60] Brown NA, Bass C, Baldwin TK, Chen H, Massot F, Carion PWC, Urban M, van de Meens AML, Hammond-Kosack KE: **Characterisation of the*****Fusarium graminearum*****-Wheat floral interaction.** *J Pathog* 2011, **626345:**1–9. 10.4061/2011/626345 PMC3335584 22567335

[61] Fellah A, Anjukandi P, Waterland MR, Williams MAK (2009). Determining the degree of methylesterification of pectin by ATR/FTIR: Methodology optimization and comparison with theoretical calculations. Carbohyd Polym.

[62] Synytsya A, Copikova J, Matejka P, Machovic V (2003). Fourier transform Raman and Infrared spectroscopy of pectins. Carbohyd Polym.

[63] Nie M, Luo J, Xiao M, Chen J, Bao K, Zhang W, Chen J, Li B (2007). Structural differences between *Fusarium* strains investigated by FT-IR Spectroscopy. Biochemistry.

[64] Barran LR, Schneider EF, Wood PJ, Madhosingh C, Miller RW (1975). Cell wall of *Fusarium sulphureum*: I. chemical composition of the hyphal wall. Biochim Biophys Acta.

[65] Bai GH, Shaner G, Ohm HM (1991). Effect of moist period on response of wheat cultivars to infection by *Fusarium graminearum*. Phytopathology.

[66] Schroeder HW, Christensen JJ (1963). Factors affecting resistance of wheat to scab caused by *Gibberella zeae*. Phytopathology.

[67] Droge-Laser W, Kaiser A, Lindsay WP, Halkier BA, Loake GJ, Doerner P, Dixon RA, Lam C (1997). Rapid stimulation of a soybean protein-serine kinase that phosphorylates a novel bZIP DNA-binding protein, G/HBF-1, during the induction of early trascripotion-dependent defenses. EMBO J.

[68] Dixon RA, Paiva N (1995). Stress-induced phenylpropanoid metabolism. Plant Cell.

[69] Briggs SP (1995). Grand unification theory in sight. Curr Biol.

[70] Orsini F, Ami D, Villa AM, Sala G, Bellotti MG, Doglia SM (2000). FT-IR microspectroscopy for microbiological studies. J Microbiol Methods.

[71] Salman A, Erukhimovitch V, Talyshinsky M, Huleihil M (2002). FTIR-spectroscopic method for detection of cells infected with herpes virues. Biopolymers.

[72] Rioux D, Oulette GB (1991). Barrier zone formation in host and nohost trees inoculated with *Ophiostoma ulmi*. I. Anatomy and histochemistry. Can J Bot.

[73] Comeau A, Langevin F, Caetano VR, Haber S, Savard ME, Voldeng H, Fedak G, Dion Y, Rioux S, Gilbert J, Martin RA, Eudes F, Scheeren PL (2011). A different path to the summit of fusarium head blight resistance in wheat: developing germplasm with a systemic approach. Plant Breed Seed Sci.

[74] DePauw RM, Knox RE, McCaig TN, Clarke FR, Clarke JM (2011). Muchmore hard red spring wheat. Can J Plant Sci.

[75] Naumann D, Helm D, Labischinski H, Giesbrecht P, Nelson WH (1991). The characterization of microorganisms by Fourier-transform infrared spectroscopy (FTIR). Modern Techniques for Rapid Microbiological Analysis.

