# Supplementary figures and images for: Synchrotron based phase contrast X-ray imaging combined with FTIR spectroscopy reveals structural and biomolecular differences in spikelets play a significant role in resistance to Fusarium in wheat
Source: BMC Plant Biol. 2015 Jan 28;15:24. doi: 10.1186/s12870-014-0357-5 (PMC4340487; doi:10.1186/s12870-014-0357-5)

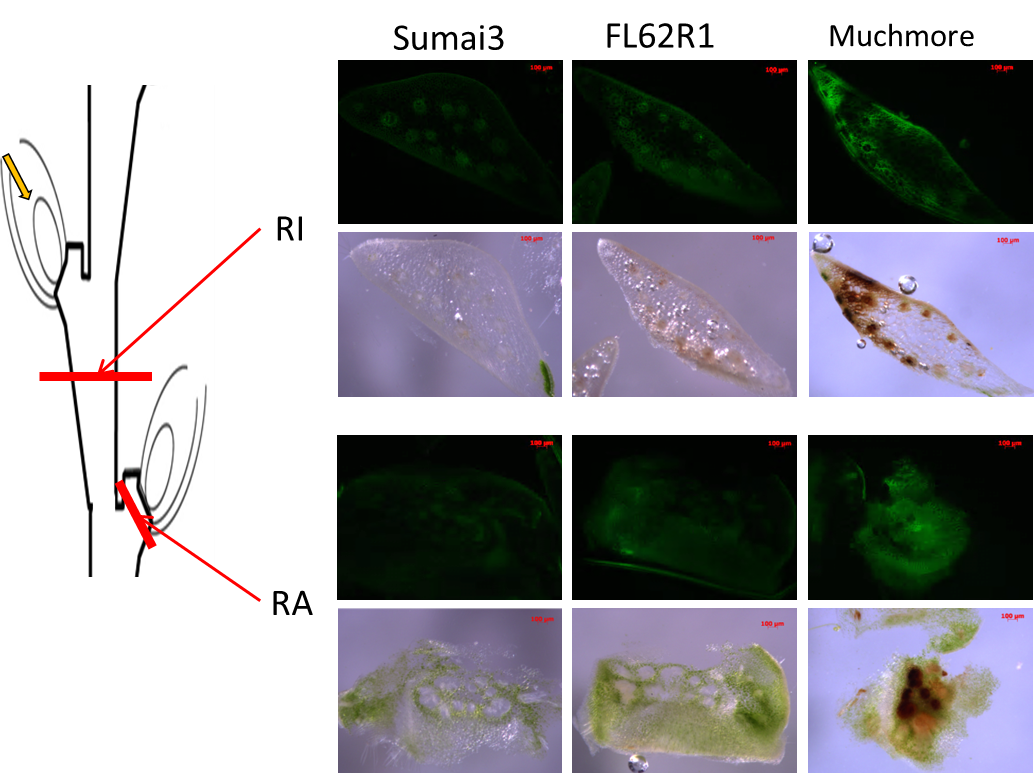

Supplement: Additional file 1: Figure S1. — Infection progress of Fg-GFP in wheat rachis at 10 DAI. A schematic illustration of wheat spike is shown in left side. For simplicity only one floret per spikelet is shown. Thick yellow arrow indicates the inoculation site. Red lines indicate the approximate position of hand sections. RI, Rachis internode; RA, Rachilla. Each sample was taken two photos under epiflurousent and white light microscope. [file 12870_2014_357_MOESM1_ESM.tiff]
