# Supplementary material for: Synchrotron based phase contrast X-ray imaging combined with FTIR spectroscopy reveals structural and biomolecular differences in spikelets play a significant role in resistance to Fusarium in wheat
Source: BMC Plant Biol. 2015 Jan 28;15:24. doi: 10.1186/s12870-014-0357-5 (PMC4340487; doi:10.1186/s12870-014-0357-5)
Supplement: Additional file 2: Tables S1–S4. — Assignments of FTIR absorption peaks (cm−1) of wheat florets at 4 days after inoculation (Table S1). Assignments of FTIR absorption peaks (cm−1) of wheat florets at 10 days after inoculation (Table S2). Assignments of FTIR absorption peaks (cm−1) of wheat rachis at 4 days after inoculation (Table S3). Assignments of FTIR absorption peaks (cm−1) of wheat rachis at 10 days after inoculation (Table S4). [file 12870_2014_357_MOESM2_ESM.docx]

**Supplemental Table 1.** Assignments of FTIR absorption peaks (cm^-1^) of wheat florets at 4 days after inoculation.

| Healthy |  |  | Infected | |  | | | Assignment | |  |
| --- | --- | --- | --- | --- | --- | --- | --- | --- | --- | --- |
| Sumai3 | FL62R1 | Muchmore | | Sumai3 | | FL62R1 | Muchmore | |  | |
| 3407.9 | 3394.3 | 3401.1 | | 3397.7 | | 3401.1 | 3404.5-3384.1 | | Stretching bands for OH groups- N-H stretching | |
| 3002.9 | 3002.9 | 3002.9 | | 3002.9 | | 3002.9 | 3002.9 | | C-H Lipid region | |
| 2928.0 | 2924.6 | 2924.6 | | 2921.2 | | 2921.2 | 2921.2 | | Alkyl C-H groups | |
| 1733.3 | 1733.3 | - | | 1733.3 | | 1733.3 | - | | C=O groups: Carbonyl compounds | |
| 1719.7 | 1712.9 | 1712.9 | | 1712.9 | | 1712.9 | - | | symmetric C=O stretch | |
| 1655.0 | 1655.0 | 1655.0 | | 1637.9 | | 1634.6 | 1637.9 | | Amide I: α-helix and β-sheet | |
| 1566.5 | 1546.1 | 1546.1 | | - | | - | - | | Amide II | |
| 1515.4 | 1518.9 | 1515.4 | | 1515.4 | | 1515.4 | 1515.4 | | Amide II | |
| 1488.2 | 1488.2 | 1484.8 | | 1484.8 | | 1484.8 | 1484.8 | | Amide II | |
| - | 1454.2 | - | | - | | - | - | | Symmetric bending modes of methyl groups I skeletal proteins | |
| 1423.5 | 1420.1 | 1423.5 | | 1420.1 | | 1420.1 | 1409.9 | | CH2 symmetric bending: cellulose | |
| 1375.9 | 1375.9 | 1375.9 | | 1382.7 | | 1379.3 | 1379.2 | | Lignin | |
| 1246.5 | 1246.5 | 1253.4 | | 1256.8 | | 1253.4 | 1253.4 | | Amide III band component of proteins-Collagen | |
| - | - | - | | 1195.5 | | 1195.5 | 1195.5 | | PO^-2^ asymmetric (phosphate) | |
| 1158.1 | 1158.1 | 1161.5 | | 1161.5 | | 1161.5 | 1161.5 | | PO^-2^ asymmetric (phosphate) | |
|  | 1079.8 |  | | - | | - | - | | Phosphates and oligosaccharides | |
| 1038.9 | 1032.1 | 1052.5 | | 1049.1 | | 1049.1 | 1049.1 | | C-O vibrations in the cellulose groups | |
| - | - | 909.6 | | 912.9 | | 912.9 | 912.9 | | C1-H bending: Xyloglucan | |
| 878.9 | 878.9 | 878.9 | | - | | - | - | |  | |
| - | - | 844.9 | | 844.9 | | 855.1 | 855.1 | | Ring vibration | |
| 827.9 | 827.9 | - | | - | | 831.3 | - | | Ring vibration | |

**Supplemental Table 2.** Assignments of FTIR absorption peaks (cm^-1^) of wheat florets at 10 days after inoculation.

| Healthy |  |  | Infected | | Assignment | | |
| --- | --- | --- | --- | --- | --- | --- | --- |
| Sumai3 | FL62R1 | Muchmore | | Sumai3 | FL62R1 | Muchmore |  |
| 3384.1 | 3373.9 | 3363.7 | | 3380.7 | 3377.3 | 3339.9 | Stretching bands for OH groups- N-H stretching |
| 2921.2 | 2917.8 | 2928.0 | | 2921.2 | 2821.2 | 2917.8 | C-H Lipid region |
| - | 2849.7 |  | | - | - | - | Alkyl C-H groups |
| - | - | - | | 1729.9 | - | 1733.3 | C=O groups: Carbonyl compounds |
| 1709.5 | 1709.5 | 1712.9 | | 1709.5 | 1709.5 | - | C=O groups: Carbonyl compounds |
| 1631.2 | 1631.2 | 1634.6 | | 1637.9 | 1631.2 | 1658.4 | Amide I |
| 1539.3 | - | 1532.5 | | - | 1539.3 | 1549.5 | Amide II |
| 1515.4 | 1515.4 | 1515.4 | | 1515.4 | 1515.4 | 1515.4 | Amide II |
| 1484.8 | 1488.2 | - | | 1488.2 | 1484.8 | - | Symmetric bending modes of methyl groups I skeletal proteins |
| 1403.1 | 1413.3 | 1406.5 | | 1420.1 | 1406.5 | 1423.5 | CH2 symmetric bending: cellulose |
| - | - | - | | - | - | 1375.9 | Lignin |
| 1338.5 | - | - | | 1338.5 | - | 1321.4 | Amide III |
| 1249.9 | 1249.9 | 1249.9 | | 1249.9 | 1249.9 | 1246.5 | PO^-2^ asymmetric (phosphate) |
| 1195.5 | - | 1209.1 | | - | 1195.5 | - | PO^-2^ asymmetric (phosphate) |
| 1158.1 | 1158.1 | - | | 1158.1 | 1158.1 | 1161.5 | Phosphates and oligosaccharides |
| 1052.5 | 1052.5 | 1055.9 | | 1052.5 | 1055.9 | 1038.9 | C-O vibrations in the cellulose groups |
| 916.4 | 912.9 | 909.6 | | 916.4 | 912.9 | 909.6 | C1-H bending: Xyloglucan |
| - | - | - | | - | 851.1 | 851.7 | Ring vibration |
| - | 831.3 | 831.3 | | 831.3 | 831.3 | - | Ring vibration |

**Supplemental Table 3.** Assignments of FTIR absorption peaks (cm-1) of wheat rachis at 4 days after inoculation.

| Healthy |  |  | Infected | Assignment | | |
| --- | --- | --- | --- | --- | --- | --- |
| Sumai3 | FL62R1 | Muchmore | Sumai3 | FL62R1 | Muchmore |  |
| 3390.9 | 3407.9 | 3407.9 | 3407.9 | 3407.9 | 3404.5 | Stretching bands for OH groups- N-H stretching |
| 3002.9 | 3019.9 | 3002.9 | - | - | - | C-H Lipid region |
| 2934.8 | 2928.0 | 2934.8 | 2934.8 | 2924.6 | 2928.0 | Alkyl C-H groups |
| 1736.7 | 1736.7 | 1736.7 | 1736.7 | 1736.7 | 1736.7 | C=O groups: Carbonyl compounds |
| 1706.1 | - | 1702.7 | 1706.1 | 1702.7 | 1709.5 | symmetric C=O stretch |
| 1658.4 | 1637.9 | 1655.0 | 1655.0 | 1655.0 | 1655.0 | Amide I: α-helix and β-sheet |
| 1566.5 | 1559.7 | 1542.7 | 1559.7 | - | - | Amide II |
| 1512.0 | 1512.0 | 1515.4 | 1515.4 | 1515.4 | 1515.4 | Amide II |
| 1484.8 | 1484.8 | 1484.8 | 1484.8 | 1484.8 | 1484.8 | Amide II |
| 1457.6 | 1457.6 | 1457.6 | 1457.6 | 1457.6 | 1457.6 | Symmetric bending modes of methyl groups I skeletal proteins |
| 1423.5 | 1423.5 | 1426.9 | 1423.5 | 1423.5 | 1423.5 | CH2 symmetric bending: cellulose |
| 1372.5 | 1375.9 | 1372.5 | 1375.3 | 1372.5 | 1375.9 | Lignin |
| 1328.2 | 1328.2 | 1324.8 | 1331.6 | 1328.2 | 1331.6 | Amide III band component of proteins-Collagen |
| 1249.9 | 1249.9 | 1249.9 | 1260.2 | 1246.5 | 1249.9 | PO^-2^ asymmetric (phosphate) |
| 1161.5 | 1161.5 | 1161.5 | 1161.5 | 1161.5 | 1161.5 | Phosphates and oligosaccharides |
| 1049.1 | 1049.1 | 1049.1 | 1049.1 | 1052.5 | 1049.1 | C-O vibrations in the cellulose groups |
| 909.6 | 909.6 | 909.6 | 909.6 | 909.6 | 909.6 | C1-H bending: Xyloglucan |
| - | - | 848.3 | 848.3 | 848.3 | 848.3 | Ring vibration |

**Supplemental Table 4.** Assignments of FTIR absorption peaks (cm^-1^) of wheat rachis at 10 days after inoculation.

| Healthy |  |  | Infected | Assignments | | |
| --- | --- | --- | --- | --- | --- | --- |
| Sumai3 | FL62R1 | Muchmore | Sumai3 | FL62R1 | Muchmore |  |
| 3407.9 | 3397.7 | 3407.9 | 3407.9 | 3404.5 | 3401.1 | Stretching bands for OH groups- N-H stretching |
| - | - | 3002.9 | - | - | - | C-H Lipid region |
| 2928.0 | 2917.8 | 2921.2 | 2924.6 | 2921.2 | 2921.2 | Alkyl C-H groups |
| - | 2849.7 | - | - | 2849.7 | - |  |
| 1736.7 | 1736.7 | 1736.7 | 1736.7 | 1736.7 | 1733.3 | C=O groups: Carbonyl compounds |
| 1706.1 | 1702.7 | 1706.1 | 1706.1 | 1709.5 | - | symmetric C=O stretch |
| 1658.4 | 1655.0 | 1655.1 | 1655.0 | 1651.6 | 1655.0 | Amide I: α-helix and β-sheet |
| - | 1597.1 | - | - | - | - |  |
| 1563.1 | 1539.3 | 1559.7 | 1563.1 | - | - | Amide II |
| - | - | - | - | 1546.1 | 1546.1 |  |
| 1512.0 | 1512.0 | 1512.0 | 1515.4 | 1515.5 | 1515.4 | Amide II |
| 1484.8 | 1484.8 | 1484.8 | 1484.8 | 1484.8 | 1484.8 | Amide II |
| 1460.9 | 1460.9 | 1460.9 | 1454.2 | 1457.6 | 1454.2 | Symmetric bending modes of methyl groups I skeletal proteins |
| 1423.5 | 1423.5 | 1423.5 | 1423.5 | 1423.5 | 1423.5 | CH2 symmetric bending: cellulose |
| 1375.9 | 1375.9 | 1375.9 | 1372.5 | 1375.9 | 1375.9 | Lignin |
| 1331.6 | 1331.6 | 1331.6 | 1331.6 | 1331.6 | 1321.4 | Amide III band component of proteins-Collagen |
| 1246.5 | 1250.0 | 12450.0 | 1246.5 | 1246.5 | 12450.0 | PO^-2^ asymmetric (phosphate) |
| - | - | - | - | 1192.1 | - |  |
| 1161.5 | 1161.5 | 1161.5 | 1161.5 | 1161.5 | 1161.5 | Phosphates and oligosaccharides |
| 1052.5 | 1049.1 | 1049.1 | 1049.1 | 1049.1 | 1049.1 | C-O vibrations in the cellulose groups |
| 909.6 | 909.6 | 909.6 | 909.6 | 909.6 | 912.9 | C1-H bending: Xyloglucan |
| - | 851.7 | 851.7 | - | 858.6 | 851.7 | Ring vibration |
